# On General and Local Disease

**Published:** 1815-06

**Authors:** Robert Kinglake

**Affiliations:** Taunton


*For the Medical and Physical Journal.*

### *On General and Local Disease;* by Dr. Kinglake.

IT is probable, that diseases of every description, however general may be their aspect, have actually in the onset a local origin. The concert of health may be variously disturbed, and whatever part may more particularly suffer from thence, the morbid influence may be propagated over the whole system. The slightest deviation from the healthy standard becomes, in the part in which it may occur, a diseased state, which may, after there exciting a disordered action, either wear itself out in that situation, or may be extended to the system, and induce an affection more or less severe, and of such character as circumstances of sympathy and visceral participation might occasion. It is difficult to conceive a state of universal disease at the commencement. Morbid causes do not at once subvert the healthy actions of life. An hurtful impression is first made on a part of the system that may have been more particularly exposed to noxious agency, and from thence it spreads and assumes a more general form. It is in this local and sympathetic way that diseases originate, extend, and diversify their character. Were the invasion of the general health to be more abrupt and universal, the attack would often prove in the first instance destructive, but its local and progressive character affords time for salutary resistance, for awakening a condition of re-action that tends to subdue disease, and to restore the healthy state.

Intimately connected as every part of the animal system is as well by continuity of general structure as by associative laws of action, the transfer or extension of a partial disease to another situation, or its equable diffusion over the frame, is a natural and inevitable operation of vital power. Local diseases are often dissipated and finally annulled by extension. The concentrated power which morbid action has, when exerted within limited boundaries, is expended, and consequently weakened when indefinitely distributed. The actions of health are strong by natural arrangement, by habit, and by associative influence; they are, therefore, not to be overcome, but by a degree of force in the morbid impression that may be capable of superinducing its own peculiar influence. Although this effect of disease may have been successful, the tenure of its ascendancy over the actions of health is at best precarious if left to itself; and, in most instances, where change of structure has not been induced, may be subdued by suitable aid. The local and general forms of diseases, may mutually originate from each other, and have commonly much reciprocity of influence. The unhealing sore may awaken morbid excitement throughout the system, whilst any ailment in the general functions may greatly aggravate the conditions of local disease. Morbid action, whether locally or generally prevailing, is capable of considerable influence in its stimulant operation in affecting the natural conditions of vital motion, but it is in the derangement it might occasion in the digestive and various secreting functions that its pernicious effect is most to be apprehended. The actions of muscles, blood-vessels, nerves, or any other part, may be disturbed without any serious injury being the result, the occurrence may not be lasting, and the natural state would quickly be restored; but, when the chylopoietic viscera, the secreting office of the liver, kidneys, and of the glandular structure at large, are involved, deep and threatning mischief is induced, and only to be remedied by so far overcoming the diseased changes in the action of the secerning vessels, as will go to reinstate the healthful condition. The morbid sympathies generated by the the failure of healthy action in the digestive and secreting functions, are various and severe, and often assume an originality of character that imposes the aspect of idiopathic affection. It is of practical importance, to make a correct discrimination on these occasions, that the remedy may be applied to the cause, instead of being unavailingly directed against an effect.

Local diseases, originating as such, or supervening a disordered state of the system, exert an hurtful influence on the digestive and secreting functions of life. Were a disease strictly local, in situation and effect, its importance would not exceed the limit of its boundaries, it might disturb and even damage the structure of the part forming its immediate seat, but that would be the extent of the mischief; the various visceral functions would not be involved, and it is obvious, that without their participation no serious danger to life could arise: but this is rarely the case. However slight the local disturbance might first be, it has a tendency to draw into its condition the vital action of the system at large, and more particularly that of the chylopoietic viscera, the glands and lymphatics. When this is the case, the distinction of local disease is unimportant; it so far assimulates the general to its own particular state as to induce universality and identity of disease. The same morbid influence on the whole system would result from any particular viscus taking on, in a disproportionate degree, any disease which might be existing in a general form. After the ailment shall have located itself, it will have infinitely more effect in disturbing the function of health, than when the cause of disease was more extenuated by being more equally diffused over the system; thus it is, that more is to be dreaded from the tendency and progress of local diseases in deranging and subverting the vital functions, than from the diseases which affect more equally, and inflict no inevitably destructive injury on any particular organ. The curative indication in all diseases, whether assuming a general or local form, must, in the first instance, be chiefly directed to the state of the vital organs. The smallest derangement in the function of either of these parts may lead to destructive consequences, and, even if that magnitude of injury should not ensue, it will tend, by vitiating the active conditions of vital power, to generate morbid susceptibilities that may become a fertile source of various distempers. Pending the existence of visceral disease, it cannot be supposed that any deviations from the healthy state in other parts can be corrected and restored. It becomes, then, a subject of the utmost moment in instituting a plan of cure for any serious disease to look well well to the conditions of the various functions of life, more particularly to those of the chylopoietic viscera. The stomach, intestines, mesenteric glands, liver, and pancreas, contribute so much severally and collectively to the support of health, that their healthful state should be an object of the first attention; hence, the pre-eminent benefit of keeping the bowels lax by suitable cathartic medicine, as it tends to keep up the biliary secretion, to prevent the mesenteric glands from being oppressed and obstructed, the diaphragm from being pressed on, the liver from being irritated, and, in connection with these benefits, it secures the incalculable advantages of healthy sympathies or associative actions throughout the system. When the stomach, bowels, and liver, duly contribute their respective parts in the digestive function; vigour and freedom from disease are the common result. If local disease has disordered this important function, it serves to shew with what detriment it acts, and that its hurtful influence should be restrained by the most efficient means; on the contrary, if the digestive function, the main-spring of health, and the sovereign remedy for local disease, should be impaired, every endeavour should be exerted to correct its ailment, and to reinstate it in its natural vigour and harmony. This may be commonly effected by duly evacuating the contents of the alimentary canal, and by observing a course of abstinence that will secure against the injuries of redundance and oppression.

*Taunton;* ROBERT KINGLAKE.

*April* 20, 1815.

